# Structural Changes Induced by Resveratrol in Monounsaturated and Polyunsaturated Phosphatidylcholine-Enriched Model Membranes

**DOI:** 10.3390/membranes13120909

**Published:** 2023-12-14

**Authors:** Rusina Hazarosova, Albena Momchilova, Victoria Vitkova, Vesela Yordanova, Aneliya Kostadinova, Miglena I. Angelova, Cedric Tessier, Philippe Nuss, Galya Staneva

**Affiliations:** 1 Institute of Biophysics and Biomedical Engineering, Bulgarian Academy of Sciences, Acad. Georgi Bonchev Str., Bl. 21, 1113 Sofia, Bulgaria; r_hazarosova@abv.bg (R.H.); albena_momchilova@abv.bg (A.M.); vyordanova@biophys.bas.bg (V.Y.); aneliakk@yahoo.com (A.K.); 2 Institute of Solid State Physics, Bulgarian Academy of Sciences, 72 Tzarigradsko Chaussee Blvd., 1784 Sofia, Bulgaria; victoria@issp.bas.bg; 3 Department of Physics, Faculty of Sciences and Engineering, Sorbonne University, 75005 Paris, France; miglena.anguelova@sorbonne-universite.fr; 4 Matière et Systèmes Complexes (MSC), CNRS UMR 7057, University Paris Cite, 75013 Paris, France; 5 Department of Psychiatry, Saint-Antoine Hospital, DMU Neuroscience, Sorbonne University, Assistance Publique-Hôpitaux de Paris (AP-HP), 75012 Paris, France; ced.tessier@gmail.com (C.T.); nuss.philippe@gmail.com (P.N.); 6 Centre de Recherche Saint-Antoine, INSERM UMRS 938, Sorbonne Université, 75012 Paris, France

**Keywords:** resveratrol, docosahexaenoic acid, oleic acid, lipid order, membrane domains, rafts

## Abstract

Resveratrol (Resv) is considered to exert a beneficial impact due to its radical scavenger, anti-microbial and anti-inflammatory properties through several mechanisms that could include its interaction with the cell plasma membrane. To address this issue, we investigated the influence of Resv on membrane lipid order and organization in large unilamellar vesicles composed of different lipids and ratios. The studied lipid membrane models were composed of phosphatidylcholine (PC) species (either palmitoyl-docosahexaenoyl phosphatidylcholine (PDPC) or palmitoyl-oleoyl phosphatidylcholine (POPC)), sphingomyelin (SM) and cholesterol (Chol). This study found that the addition of Resv resulted in complex membrane reorganization depending on the degree of fatty acid unsaturation at the *sn-2* position, and the Lipid/Resv and SM/Chol ratios. Resv rigidified POPC-containing membranes and increased liquid-ordered (L_o_) domain formation in 40/40/20 POPC/SM/Chol mixtures as this increase was lower at a 33/33/34 ratio. In contrast, Resv interacted with PDPC/SM/Chol mixtures in a bimodal manner by fluidizing/rigidifying the membranes in a dose-dependent way. L_o_ domain formation upon Resv addition occurred via the following bimodal mode of action: L_o_ domain size increased at low Resv concentrations; then, L_o_ domain size decreased at higher ones. To account for the variable effect of Resv, we suggest that it may act as a “spacer” at low doses, with a transition to a more “filler” position in the lipid bulk. We hypothesize that one of the roles of Resv is to tune the lipid order and organization of cell plasma membranes, which is closely linked to important cell functions such as membrane sorting and trafficking.

## 1. Introduction

Resveratrol (Resv, trans-3,5,4′-trihydrostilbene) is a fat-soluble substance, and a member of a group of plant-derived compounds called polyphenols found in red wine and in several plants such as grapes, raspberries, mulberries, blueberries and peanuts [1]. It is also a natural phytoalexin (a compound with antibiotic properties), produced by a wide variety of plants to protect them against pathogens and/or environmental stress [2]. Resveratrol has attracted attention in the context of the so-called “French paradox”, the epidemiological observation that the consumption of red wine is associated with a reduced rate of coronary heart disease [3]. Many clinical and experimental studies have demonstrated the pleiotropic effects of Resv. Growing evidence suggests that polyphenols, such as Resv, possess a range of beneficial biological properties, including antioxidant, anti-inflammatory, cardioprotective, antitumor, neuroprotective, chemopreventive, anti-diabetic, anti-obesity and anti-aging properties [4]. Antiglycation properties of Resv have been reported in recent studies [5,6]. This compound can effectively prevent complications related to diabetes mellitus, such as renal impairment [7], ocular damage [8] and ear injuries [9]. Numerous publications have also confirmed that Resv is a free-radical scavenger [10,11,12]. This antioxidant activity is linked to the existence of three hydroxyl groups within its chemical structure. Resveratrol inhibits excessive production of ROS, an abnormal distribution of mitochondria and lipid peroxidation, particularly LDL oxidation [11,12]. The anti-inflammatory properties of Resv have been extensively documented. It was shown to suppress IL-6 transcription and translation [13] and reduce the expression of inflammatory mediators such as TNF-α and IL-8 [14]. Neuroprotective effects have also been demonstrated, in particular in murine animal models, reducing neurodegeneration and facilitating memory recovery following toxic exposure or vascular damage [15,16]. Resveratrol has been found to have multiple biological partners, including sirtuins, brain-derived neurotrophic factor (BDNF), endocannabinoids and polyunsaturated fatty acids (PUFAs) [17].

The clinical effectiveness of Resv as an oral supplement was subject to question because of problems with its bioavailability. The compound has a short half-life of 1.5 h as it is rapidly absorbed via active transport by intestinal epithelial cells and degraded in the liver [18]. To overcome this issue, nanoparticles and nanostructured lipid carriers containing Resv or prodrugs have been proposed to increase its bioavailability [19,20].

Several studies have investigated the potential biophysical mechanism of action of this substance in contributing to the aforementioned biological functions, owing to its lipophilic properties and capability to intercalate into cell membranes. The current study follows a molecular structural arrangement approach, assuming that the interaction of Resv within the lipid bulk of the membrane could potentially account for some of its biological properties. It is interesting to speculate that compounds such as Resv may act to facilitate optimal membrane lipid organization to enable membrane sorting, trafficking and cell signaling [21]. The organization of lipids within membranes is critical, in particular the segregation of lipids into lateral domains, particularly those enriched in cholesterol and sphingolipids, called *rafts*. To mimic this process, model membranes have been developed because they can form liquid-ordered (L_o_) domains (lipid rafts), composed predominantly of SM and Chol, surrounded by a liquid-disordered (L_d_) PC-enriched matrix [22,23].

To examine the effect of Resv on lipid membrane organization, several model membrane mixtures have been investigated using different biophysical methods. Di-palmitoyl-phosphatidylcholine (DPPC), di-miristoyl-phosphatidylcholine (DMPC), egg yolk PC (eggPC), SM and Chol, binary eggPC/Chol and ternary eggPC/SM/Chol mixtures have been studied by Longo et al. [24] and Neves et al. [25,26] using various analytical techniques, such as ESR, DSC, X-ray scattering analysis, derivative spectrophotometry, fluorescence quenching and fluorescence anisotropy. Resv was shown to be a surface-adsorbed molecule affecting the structure and the dynamics of the DPPC lipid chains in the gel phase, but not in the fluid phase. It could also act as a cholesterol mimetic on the lipid membrane structure modulating membrane fluidity. In addition, Resv was shown to induce phase separation, promoting the formation of lipid domains in eggPC, eggPC/Chol and eggPC/SM/Chol bilayers. In this regard, it was shown to influence the activity of some membrane-located proteins such as Na^+^/K^+^ and Ca^2+^ ATPases in human erythrocyte membranes [27].

To our knowledge, no study has examined the effect of Resv on lipid mixtures comprising PUFA-containing phospholipids (PLs). In the present study, two PCs differing in their degree of fatty acid (FA) length and unsaturation will allow us to disentangle the effect of Resv in model lipid membranes as a function of the degree of *sn-2* chain length and unsaturation. For this, we have included in our model lipid mixtures of either POPC (palmitoyl-oleoyl phosphatidylcholine) or PDPC (palmitoyl-docosahexaenoyl phosphatidylcholine). These lipids are characterized by having an identical head group and *sn-1* FA chain (palmitic acid, C16) but a different FA at the *sn-2* position. POPC contains oleic acid (OA, C18:1 monounsaturated FA), while PDPC contains docosahexaenoic acid (DHA, C22:6 polyunsaturated FA) belonging to the omega-3 FA family. To mimic the physiological conditions of the external layer of plasma membranes of eukaryotic cells, lipid mixtures composed of three lipid classes, sphingolipids, cholesterol and glycerophospholipids (POPC or PDPC), alone or in combination, were examined. The Resv effect on vesicle size at physiological temperature was assessed by dynamic light scattering (DLS). The thermal behavior of the Resv-treated membrane lipid models was investigated by fluorescence spectroscopy. The estimation of L_o_ domain sizes was performed by adding TEMPO to DPH-containing L_o_/L_d_ membranes, since TEMPO is able to more effectively quench the DPH fluorescence originating from the L_d_ phase. Laurdan fluorimetry was used to provide insights on the quantity of water surrounding the PL glycerol backbone, thus shedding light on the membrane structural organization at the hydrophilic/hydrophobic interface.

## 2. Materials 

The chemicals POPC (1-palmitoyl-2-oleoyl-*sn*-glycero-3-phosphocholine), PDPC (1-palmitoyl-2-docosahexaenoyl-*sn*-glycero-3-phosphocholine), SM (egg yolk sphingomyelin) and Chol (cholesterol) were purchased from Avanti Polar Lipids. The fluorescence markers Laurdan (2-(dimethylamino)-6-dodecanoylnaphthalene) and DPH (1,6-Diphenyl-1,3,5-hexatriene) and radical marker TEMPO ((2,2,6,6-Tetramethylpiperidin-1-yl)oxyl or (2,2,6,6-tetramethylpiperidin-1-yl)oxidanyl) were purchased from Sigma-Aldrich. Resveratrol (trans-3,5,4′-trihydrostilbene) was bought from Santa Crus Biotechnology. 

## 3. Methods

### 3.1. Liposome Preparation

Liposomes (1 mM Lipid) were formed by using different concentrations of Resv (50 µM (Lipid/Resv 20:1), 100 µM (Lipid/Resv 10:1), 200 µM (Lipid/Resv 5:1) and 500 µM (Lipid/Resv 2:1)). They were prepared through evaporation of the solvents from the lipid solution until dryness by using a stream of nitrogen; the lipid film was then left under vacuum (overnight) to remove all traces of the organic solvent. The dried lipid film was dispersed with 10 mM Hepes buffer (150 mM NaCl, pH 7.4), and the mixture was vortexed and sonicated to yield multilamellar vesicles (MLVs). Then, the lipid suspensions were equilibrated at a temperature above T_m_ (miscibility transition temperature; room temperature for PC liposomes and 60 °C for PC/Chol and PC/SM/Chol ones) for 10 min, vortexed and sonicated for 1 min, and then, cooled down in ice for 10 min. This procedure was repeated 3 times. After the lipid (Lipid/Resv) mixing procedure, the MLV suspensions were extruded through polycarbonate filters with a pore diameter of 800 and 100 nm to form large unilamellar vesicles (LUVs) [28]. 

### 3.2. Laurdan Fluorescence Measurements

Lipid/Resv mixtures were Laurdan-labeled (100:1 Lipid/Laurdan) in the initial organic solvent. Fluorescence emission spectra were recorded by a Hitachi Fluorescence Spectrophotometer F-7000 (Hitachi High-Technologies Corporation, Tokyo, Japan), equipped with a xenon lamp with excitation and emission slits set to 5 nm. The temperature in a cuvette containing a magnetic stir bar was measured by using a platinum thermometer and maintained under the control of a water-circulating bath. The emission spectra of Laurdan were recorded between 390 and 600 nm, whereas the samples were excited at 355 nm. The generalized polarization (GP) at 355 nm excitation was calculated using fixed emission wavelengths of 440 nm and 490 nm (GP_ex_ = (I_440_ − I_490_)/(I_440_ + I_490_)), where I_440_ and I_490_ are emission intensities at the characteristic wavelengths of the ordered phase (440 nm) and the disordered phase (490 nm) [29]. The fluorescence measurements [30] were performed at temperatures from 27 to 57 °C with an interval of 5 °C. 

### 3.3. DPH-TEMPO Fluorescence Spectroscopy

The appropriate volumes of organic solutions of lipids were mixed with the fluorescent DPH probe. The final lipid concentration in the cuvette was 500 µM as the molar ratio of lipids to DPH was 1000:1. The fluorescence quenching of DPH by the quencher TEMPO (2 mM) at 37 °C allows for the identification of the L_o_ domain fraction. TEMPO dissolved in ethanol was added to the samples, defined as F samples. The same volume of ethanol was added to the samples that did not contain TEMPO, denoted as F_o_ samples. The samples were incubated at 37 °C for 10 min, and then, the L_o_ domain sizes were calculated using the ratio Q = F/F_o_; the higher the F/F_o_ ratio, the higher the average L_o_ domain size.

### 3.4. Dynamic Light Scattering (DLS) Size Measurements

The hydrodynamic diameter (D_h_, nm), herein also named vesicle size, and the polydispersity index (PDI) were measured by DLS experiments using Zetasizer Advance Series Instrument (Malvern Analytical, Malvern, UK). The D_h_ and PDI of control PC/SM/Chol (40/40/20 and 33/33/34) and Resv-containing LUVs were collected at 37 °C. For the measurements, 1 mL of 500 µM liposome suspension in 10 mM Hepes buffer (150 mM NaCl, pH 7.4) was loaded into the glass cuvette. Six measurements were performed per sample to calculate the standard deviation of the obtained values.

## 4. Results 

### 4.1. Laurdan Fluorescence Spectroscopy

Due to the difference in solvent relaxation of Laurdan as a function of its phospholipid environment, the GP value is usually considered a proxy for the mode of lipid packing in the membrane. A positive GP value (max = 1) describes membranes in an ordered state (gel (L_β_) and liquid-ordered (L_o_)), while a negative value (max = −1) is assigned to those in a liquid-disordered state (L_d_). However, realistically, in experiments, the values range from −0.3 to 0.6 for both single- and multi-component lipid vesicles [31].

#### 4.1.1. Pure POPC and PDPC Liposomes

**Figure 1:**
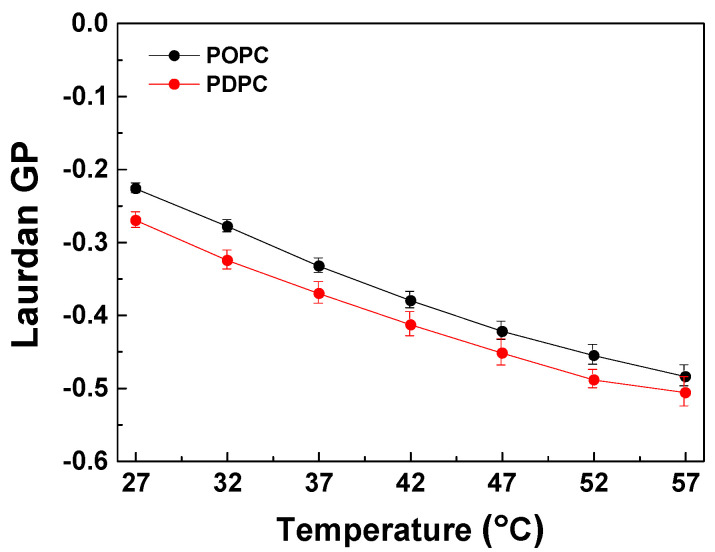
Laurdan GP values as a function of temperature (27–57 °C) in POPC and PDPC LUVs. SDs are calculated from 2–3 experiments with 6 measurements per point.

In pure PC liposomes (POPC and PDPC), the temperature increase (in the range of 27 to 57 °C) led to a gradual decrease in the GP values. As expected, the liquid-disordered state of both types of PC samples was confirmed by negative GP values (Figure 1). Over the temperature range from 27 to 57 °C, the GP value of PDPC vesicles was consistently lower than that of POPC vesicles, due to the greater degree of unsaturation of the DHA fatty acid at the *sn-2* position in PDPC. The change in GP (ΔGP) at the studied temperature range for POPC is higher compared to PDPC, suggesting slightly higher thermal stability of DHA-rich PC mixtures.

**Figure 2:**
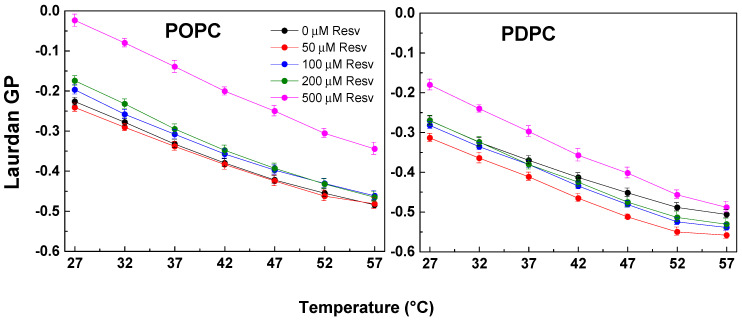
Laurdan GP values as a function of temperature (27–57 °C) in POPC and PDPC LUVs, with and without Resv (50, 100, 200 and 500 μM corresponding to 20:1, 10:1, 5:1 and 2:1 Lipid/Resv ratios). An ordering effect of Resv in POPC vesicles was observed, while both fluidizing and ordering effects were found for PDPC ones depending on Resv concentration. SDs are calculated from 2–3 experiments with 6 measurements per point.

The addition of Resv in POPC membranes resulted in increased packing. In the PDPC samples, low and medium Resv doses increased membrane fluidity, whereas at the highest Resv concentration studied, higher GP was measured for PDPC bilayers. This bimodal effect in PDPC on low versus high Resv concentrations was observed in most of the studied samples (Figure 2).

#### 4.1.2. Binary PC/Chol Mixtures 

As expected, Chol addition was associated with increased GP values. As shown by Almeida et al. [32], using several photophysical methodologies, binary and ternary POPC/Chol and POPC/SM/Chol mixtures can exhibit L_o_/L_d_ phase separation. For POPC/Chol mixtures, L_o_ and L_d_ phases coexist for mixtures comprising from 10 to 40 mol% Chol. Applied to our results, this means that at low (25 mol%) Chol concentrations, L_o_ and L_d_ coexist for both PC mixtures as the measured GP is averaged by both phases. In contrast, at an equimolar PC/Chol ratio, only L_o_ organization is attested by highly positive GP values. 

**Figure 3:**
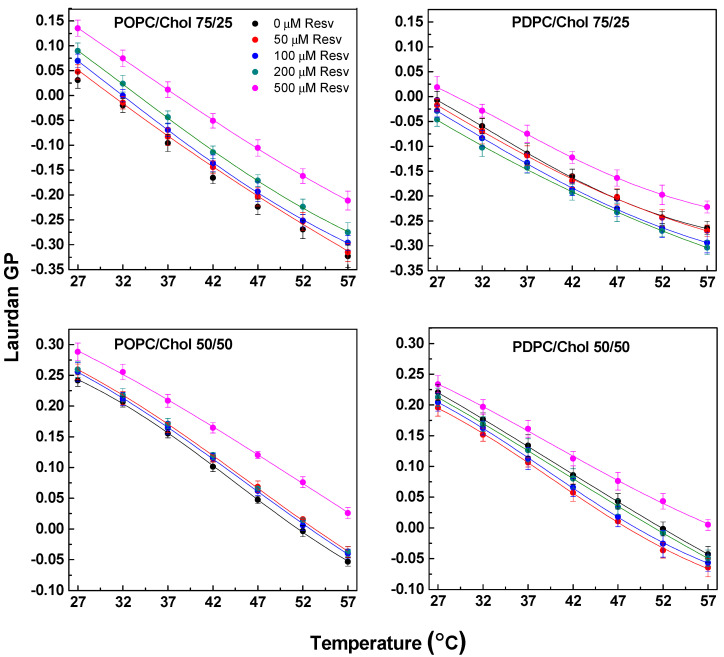
Laurdan GP values as a function of temperature in PC/Chol mixtures (75/25 and 50/50). Control condition and effect of various Resv concentrations (50, 100, 200 and 500 μM corresponding to 20:1, 10:1, 5:1 and 2:1 Lipid/Resv ratios) are displayed using different colors. An ordering effect of Resv in POPC/Chol mixtures (left-hand graphs) was observed, while both fluidizing and ordering effects were found for PDPC/Chol mixtures (right-hand graphs). SDs are calculated from 2–3 experiments with 6 measurements per point.

Higher GP was measured in POPC mixtures compared with PDPC ones. Of note, at the highest Chol content, the ΔGP values of both POPC and PDPC mixtures are comparable, indicating that Chol attenuates the disordering effect of DHA. In line with the findings for Resv addition in pure POPC, Resv induced rigidification in binary POPC/Chol mixtures. In contrast to POPC mixtures and consistent with pure PDPC LUVs, a bimodal effect was still observed after the addition of Resv in PDPC/Chol mixtures. For both studied PDPC/Chol mixtures, maximum fluidization was observed for the 50 µM Resv sample. In contrast, the addition of 500 µM of Resv resulted in rigidification of these mixtures (Figure 3).

**Figure 4:**
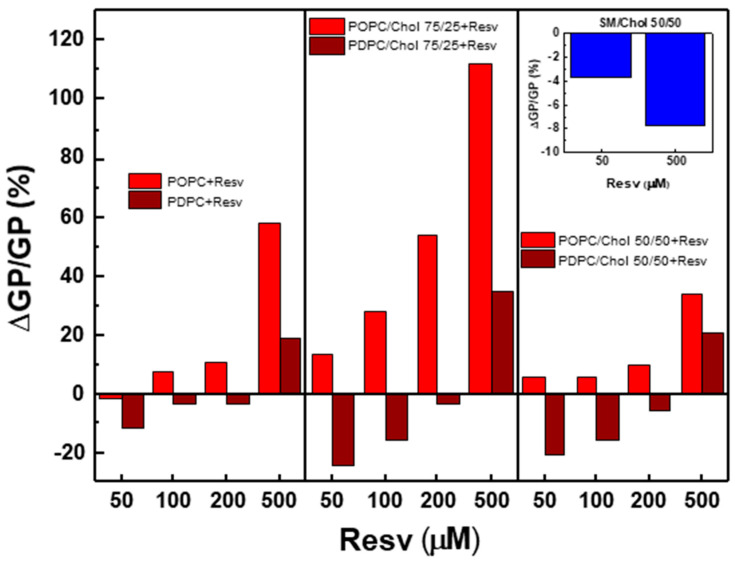
ΔGP/GP (%) values at varying Resv concentrations for LUVs made up of PC, PC/Chol (75/25 and 50/50) and SM/Chol (50/50) at 37 °C. The blue bars represent the SM/Chol LUVs.

To evaluate the Resv effect on the lipid phase states, we calculated the relative GP change, ΔGP/GP (in %), where ΔGP = GP_Resv_ − GP_0_. Here, GP_Resv_ denotes the value after Resv addition, and GP_0_ stands for the same sample free of Resv at 37 °C (Figure 4). Positive ΔGP values correspond with the ordering effect of Resv and negative ΔGP values indicate fluidization.

The addition of Resv to single-component PC LUVs had little or no effect up to 200 µM. At 500 µM, Resv exhibited a significant ordering effect on POPC and a nearly three times lower but significant effect on PDPC. The non-monotonic effect of Resv on PDPC is particularly evident at 50 µM and 500 µM (Figure 4).

The bimodal effect of Resv on binary PDPC/Chol mixtures is more pronounced than in pure PDPC bilayers. However, in Chol-enriched mixtures (50/50), the rigidifying effect of 500 µM Resv on both types of PC mixtures is weak, indicating a dampening effect of Chol on membrane ordering.

#### 4.1.3. Pure SM Vesicles

**Figure 5:**
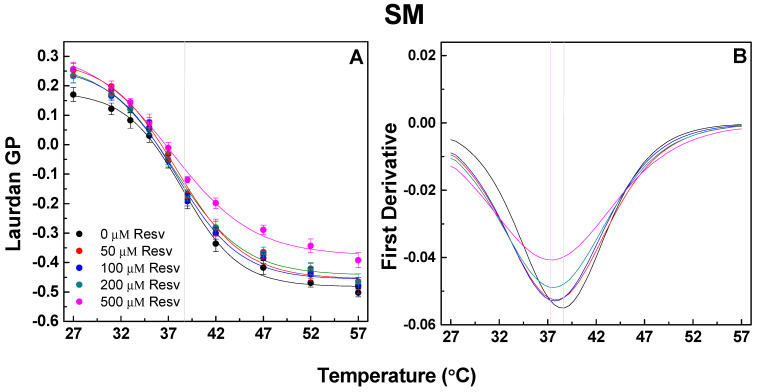
Laurdan GP values as a function of temperature in SM vesicles and Resv addition (50, 100, 200 and 500 μM corresponding to 20:1, 10:1, 5:1 and 2:1 Lipid/Resv ratios). (**A**) L_β_/L_d_ phase transition is evidenced in the thermal GP sigmoid curve. (**B**) First derivative of the GP sigmoid curve fitted by Lorentzian distribution shows differences in center peak shift and width of the studied mixtures. SDs are calculated from 2–3 experiments with 6 measurements per point.

As anticipated, egg sphingomyelin vesicles exhibit a gel (L_β_)-to-L_d_ phase transition upon heating, with a sigmoid thermal curve displaying an inflection point at 38.5 °C (Figure 5). Upon Resv addition, a dose-dependent GP increase is observed. The first derivative of the GP sigmoid curve, which is fitted with a Lorentzian distribution, reveals a shift in the center peak towards lower temperatures and an enlargement in peak width with an increase in Resv dosage (Table 1). The SM L_β_/L_d_ phase transition temperature is thus decreased after Resv addition. 

#### 4.1.4. Binary SM/Chol (50/50 mol/mol) Mixture

**Figure 6:**
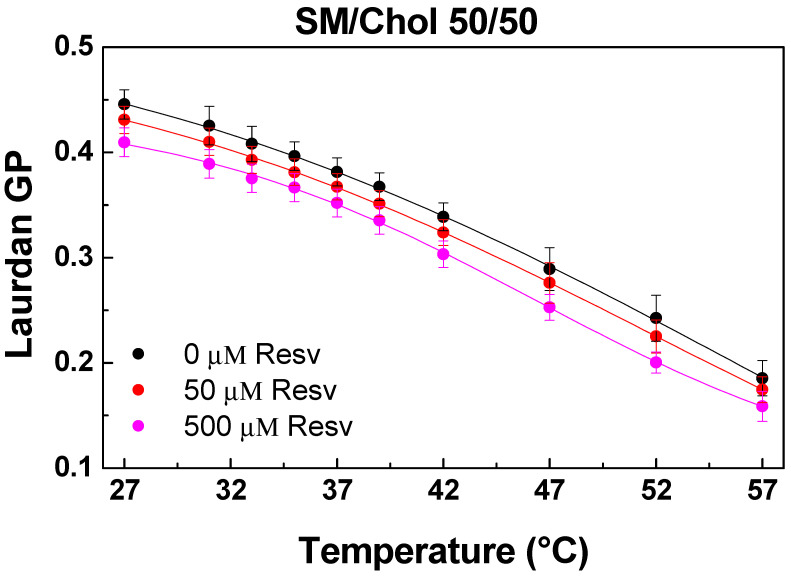
Laurdan GP values as a function of temperature in SM/Chol (50/50) vesicles as well as Resv addition (50 and 500 μM corresponding to 20:1 and 2:1 Lipid/Resv ratios). Positive and high GP values indicate L_o_ phase organization. A fluidizing effect of Resv on the L_o_ phase is observed. SDs are calculated from 2–3 experiments with 6 measurements per point.

As expected, the addition of Chol to SM resulted in positive and further-increased GP values, corresponding to the L_o_ phase organization of the mixture within the temperature range (27–57 °C), during which a decrease in GP was observed with increasing temperature (Figure 6). Resv addition led to lower GP values in a dose-dependent manner, indicating the fluidizing effect of Resv on the L_o_ phase. Surprisingly, Resv was able to fluidize the SM/Chol L_o_ phase and to rigidify the POPC/Chol one (50/50 mol/mol) (Figure 4), implying a specific conformation of Resv in SM versus POPC bilayers.

#### 4.1.5. Ternary PC/SM/Chol Mixtures (PC/SM Equimolar Ratio with Variable Chol Concentration (10, 20 or 34 mol%)

**Figure 7:**
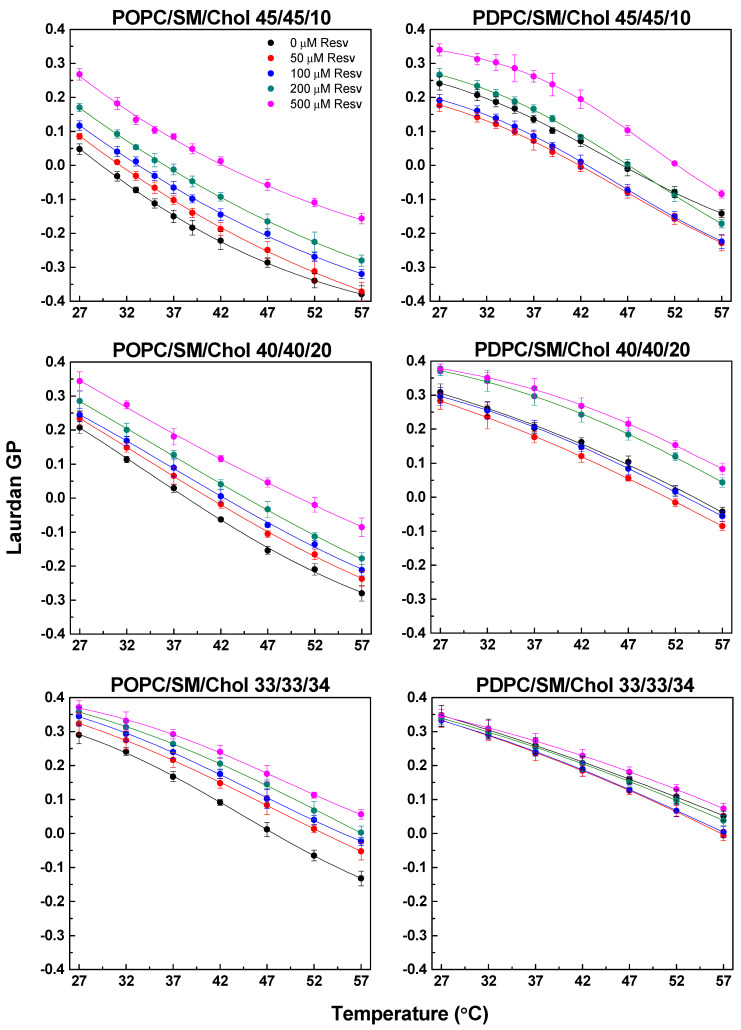
Laurdan GP values in ternary POPC/SM/Chol (left-hand graphs) and PDPC/SM/Chol (right-hand graphs) mixtures with and without Resv (50, 100, 200 and 500 μM corresponding to 20:1, 10:1, 5:1 and 2:1 Lipid/Resv ratios). SDs are calculated from 2–3 experiments with 6 measurements per point.

The ternary mixtures studied here are known to exhibit a L_o_/L_d_ phase separation at 25 °C and 37 °C [32]. We report higher GP values of PDPC mixtures corresponding to stronger lipid packing (Figure 7). 

The addition of Resv in ternary membranes resulted in changes similar to those observed in pure and binary mixtures. Rigidification of all Chol-containing ternary POPC membranes was observed in the presence of the polyphenol. In parallel, membrane fluidization was evidenced after the addition of 50 and 100 µM Resv in PDPC mixtures, whereas rigidification was observed upon 200 and 500 µM Resv addition in mixtures containing 10 and 20 mol% Chol. This Resv-related ordering effect was strongly attenuated in the ternary PDPC equimolar mixtures upon the addition of 500 µM Resv compared to the mixtures with lower Chol concentrations.

**Figure 8:**
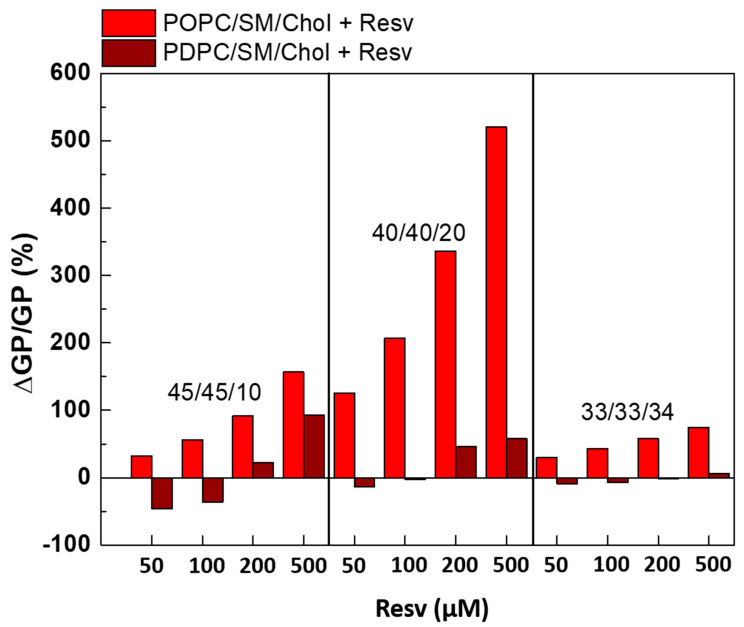
ΔGP/GP (%) as a function of Resv concentration for LUVs made of PC/SM/Chol (45/45/10, 40/40/20 and 33/33/34) mixtures calculated at 37 °C.

POPC-containing ternary mixtures exhibited the most significant ordering effect upon Resv addition (Figure 8). The strength of the effect increased gradually with the polyphenol concentration. As mentioned above, all studied ternary mixtures exhibited a L_o_/L_d_ phase coexistence. The present results confirm the averaged (L_o_ + L_d_) ordering effect of Resv on POPC/SM/Chol membranes. This effect was astonishingly more pronounced in ternary POPC/SM/Chol mixtures upon 500 µM Resv addition compared to the binary POPC/Chol mixtures (Figure 4). Such a striking difference was not observed when comparing ternary PDPC/SM/Chol mixtures with binary PDPC/Chol ones. 

### 4.2. DPH-TEMPO Fluorescence Spectroscopy of Ternary Mixtures

#### 4.2.1. Ternary PC/SM/Chol Mixtures

**Figure 9:**
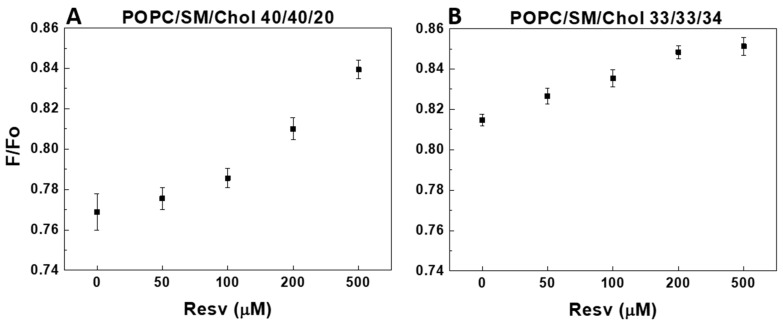
Resv’s effect on DPH fluorescence quenched by TEMPO. F/F_o_ as a function of Resv concentration in POPC/SM/Chol LUVs. F is the residual fluorescence when DPH is quenched by TEMPO, whereas F_o_ is the fluorescence from DPH without TEMPO, both measured at 37 °C. Effect of Chol concentration: F/F_o_ derived from 40/40/20 POPC/SM/Chol LUVs (**A**) and F/F_o_ derived from 33/33/34 POPC/SM/Chol LUVs (**B**). The error bars are ± SD of at least four independent measurements.

**Figure 10:**
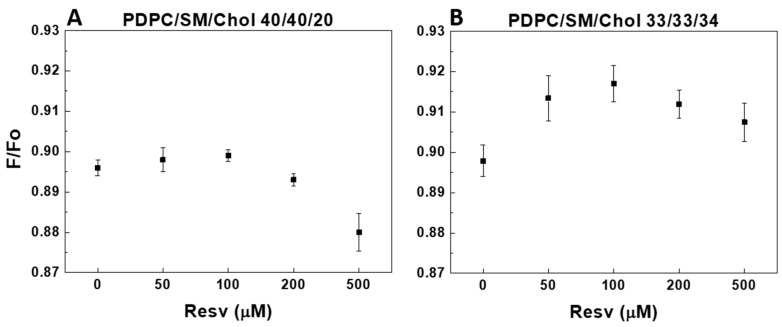
Resv’s effect on DPH fluorescence quenched by TEMPO. F/F_o_ as a function of Resv concentration in PDPC/SM/Chol LUVs. F is the residual fluorescence when DPH is quenched by TEMPO, whereas F_o_ is the fluorescence from DPH without TEMPO, both measured at 37 °C. Effect of Chol concentration: F/F_o_ derived from 40/40/20 PDPC/SM/Chol LUVs (**A**) and F/F_o_ derived from 33/33/34 PDPC/SM/Chol LUVs (**B**). The error bars are ± SD of at least four independent measurements.

POPC/SM/Chol and PDPC/SM/Chol membranes exhibited L_o_/L_d_ phase separation at physiological temperature [32,33]. The fluorophore DPH partitioned evenly between the L_o_ and L_d_ domains [34]. The small nitroxide-containing molecule TEMPO can diffuse more easily in L_d_ domains than in L_o_ ones. Hence, DPH quenching by TEMPO is stronger in L_d_ domains than in L_o_ ones. Fluorescence resonance energy transfer (FRET) emerged as an efficient tool for measuring distances between 30 and 80 Å [35]. TEMPO quenching is an attractive candidate as a sub-30 Å distance ruler for small ensemble/single-molecule measurements. Effective nitroxide quenching range (Rc) is reported to be between 6 and 12 Å [34,36] depending on the fluorophore used and decreases by 50% at about Ro (4Rc), thus sensing distances from 6 to 48 Å [37]. The calculation of exact domain size from FRET/quenching is challenging as it depends on a lot of parameters, such as acceptor concentration, the partition of donor and acceptor between ordered and disordered domains, domain shape, etc. However, comparing F/F_o_ in two types of L_o_/L_d_-presenting mixtures makes it possible to qualitatively determine in which mixture the L_o_ nanodomain formation is larger. Figure 9 and Figure 10 illustrate the capacity of TEMPO to quench DPH, expressed as the F/F_o_ ratio. The higher the F/F_o_ ratio, the lower TEMPO’s efficacy to quench DPH. This, in turn, defines a higher affinity for L_o_ nanodomain formation in the mixture. F/F_o_ is given as a function of Resv concentration and the degree of fatty acid for 20 and 34 mol% Chol in the PC/SM/Chol mixtures at 37 °C. 

Upon the addition of Resv, both ternary POPC mixtures demonstrate an increase in L_o_ domain formation (Figure 9). Of note, the control F/F_o_ values differ between the two PC mixtures (Figure 9 and Figure 10), revealing larger L_o_ domain formation in the ternary PDPC membranes compared to POPC-containing bilayers. For the highest Chol content in ternary PDPC mixtures, the F/F_o_ increase is bimodal, peaking at 100 µM Resv (Figure 10B), similar to the structural effect induced by Resv in all PDPC-containing mixtures. At the lower Chol concentration studied, this non-monotonic effect of Resv is less pronounced (Figure 10A). 

Upon the addition of Resv, both ternary POPC mixtures demonstrated an increase in L_o_ domain formation as F/F_o_ reached up to 85% (Figure 9). Of note, the control F/F_o_ values differ between the two PC mixtures (with F/F_o_ of 0.769 and 0.815 for POPC at 20 and 34 mol% Chol, respectively (Figure 9A,B) versus F/F_o_ of 0.896 and 0.898 (Figure 10A,B) for PDPC), indicative of larger L_o_ domain formation in the ternary PDPC mixtures. The addition of 500 µM Resv in ternary PDPC mixtures resulted in a L_o_ domain formation decrease for the 20 mol% Chol mixture, whereas an overall increase was observed for the equimolar mixture. For the 34 mol% Chol mixtures, the increase was bimodal, peaking at 100 µM Resv, similar to the structural effect induced by Resv in all PDPC-containing mixtures. In the 20 mol% Chol mixtures, a small increase in L_o_ domain formation occurs initially (up to 100 µM Resv), followed by a sharp decrease for 500 µM Resv.

#### 4.2.2. Calculation of the Domain Radius in Ternary PC/SM/Chol Mixtures upon Resv Addition

**Figure 11:**
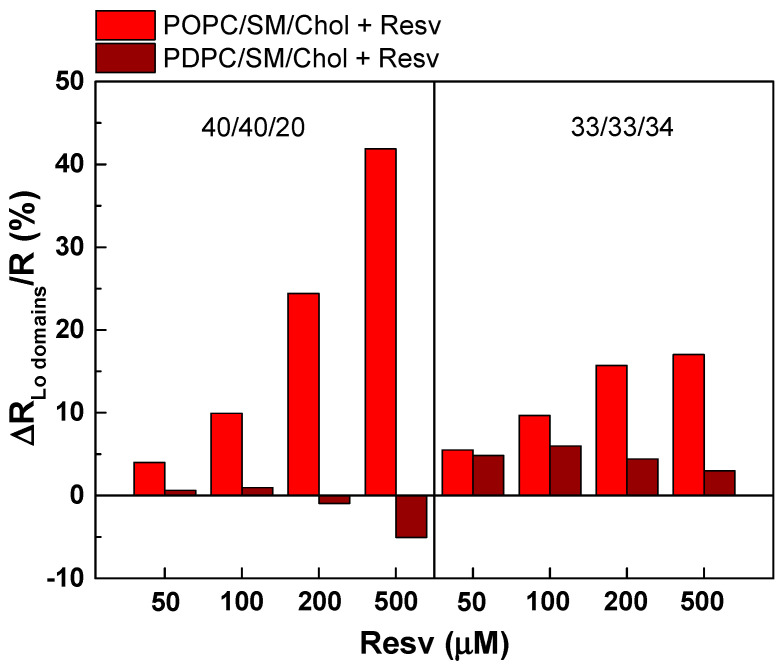
Resv’s effect on L_o_ domain radius. ΔR/R (%) as a function of Resv concentration for LUVs made of ternary PC/SM/Chol (40/40/20 and 33/33/34) mixtures at 37 °C.

The domain radii R (in Å) in the ternary mixtures were calculated following the equation R = R_o_ [(Q − Q_PC_)/(Q_SM/Chol_ − Q_PC_)] [37], where Q_PC_ and Q_SM/Chol_ are the residual DPH fluorescences of the "pure" lipid phases, PC as a model of the L_d_ phase, and SM/Chol (50/50) as a model of the L_o_ phase. The domain radii for both PC/SM/Chol mixtures at 37 °C as a function of Resv concentration were calculated and are summarized in Table 2. The baseline F/F_o_ values for pure PC vesicles were 0.60 for POPC versus 0.58 for PDPC, whereas the upper-limit F/F_o_ value for 50/50 SM/Chol vesicles was 0.93. At the lowest Chol concentration, the radius of the L_o_ domains was approximately half the size in POPC mixtures (24 Å) compared to the PDPC ones (43 Å). The increase in the Chol content resulted in a further increase in domain size for POPC in contrast to PDPC (Figure 11). Upon Resv addition, the L_o_ domain size in POPC mixtures increased with increasing Resv, whereas no change was observed in PDPC mixtures.

### 4.3. Hydrodynamic Diameter and Polydispersity Index Measurements of Ternary PC/SM/Chol LUVs upon Resv Addition

**Figure 12:**
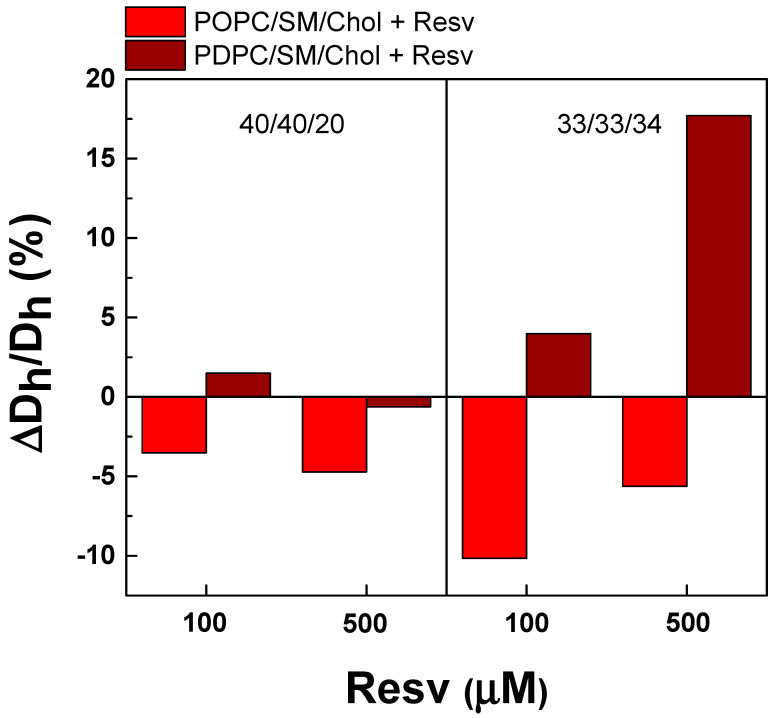
Resv’s effect on hydrodynamic diameter (D_h_) of LUVs. ΔD_h_/D_h_ (%) as a function of Resv concentration for LUVs made of ternary PC/SM/Chol (40/40/20 and 33/33/34) mixtures at 37 °C.

As DPH-TEMPO quenching could depend on the membrane curvature, we carried out DLS experiments to measure the hydrodynamic diameter (D_h_, nm) and polydispersity index (PDI) of LUVs (Table 3 and Table 4). For the LUVs without Resv, narrow monomodal size distributions were obtained for both types of PC ternary mixtures (Table 3). The PDIs for POPC/SM/Chol were slightly lower compared to PDPC/SM/Chol mixtures (Table 4). This shows that the extruded POPC/SM/Chol mixtures represented slightly higher monodispersity compared to the PDPC ones. Resv addition (100 and 500 µM) slightly decreased this monodispersity for both types of PC ternary mixtures as this decrease was stronger for PDPC ternary mixtures and for higher Chol molar ratios (Table 4). The presence of Resv decreased the averaged D_h_ for POPC ternary mixtures (33/33/34). In contrast, the higher Resv concentration induced the formation of vesicles with larger sizes in PDPC ternary mixtures (Figure 12).

## 5. Discussion

The results of this study demonstrate that Resv could be incorporated into the studied lipid mixtures exhibiting L_d_, L_o_ or L_o_/L_d_ phase coexistence. The addition of Resv resulted in specific fluidization/rigidification behavior, depending on the length and unsaturation of the *sn-2* fatty acid bonded to PC and Resv concentration. Resv induced an increased lipid order for all POPC-containing membranes. In PDPC membranes, Resv elicited a bimodal response depending on the concentration added. The L_d_ phase was fluidized with the lowest concentration of Resv, but rigidification was induced with the highest concentration. Additionally, for the SM/Chol equimolar mixture, Resv addition resulted in a decrease in GP, indicating its fluidizing effect on the L_o_ phase. Focusing on Chol’s effect in ternary mixtures, a difference was observed between POPC and PDPC mixtures. At 37 °C, Resv addition led to an increase in L_o_ domain formation for both POPC/SM/Chol mixtures. For PDPC, the addition of 500 µM Resv resulted in a decrease in L_o_ domain formation for the 20 mol% Chol mixture, whereas an overall increase was observed for 34 mol%. 

Resveratrol is poorly soluble in water, thus explaining its high affinity for the cell plasma membrane. While some studies show a location of Resv around the hydrophilic polar head region of lipids [38,39,40], others indicate a deeper positioning around non-polar hydrophobic fatty acid tails of the lipid bilayer [25,26,41,42,43]. Ghellinck at al., for instance, have shown on saturated PC membranes that Resv preferentially distributes into the headgroup region and locally changes the headgroup tilt angle to a more upright orientation [40]. Molecular docking studies also have identified the preferred geometries and energetics for the interaction of Resv within the polar headgroups of phospholipids at the hydrophilic/hydrophobic interface with the membrane. The phenolic hydroxyl groups of Resv are hydrogen bond donors and form an anchoring moiety with the corresponding acceptor oxygens in the DPPC molecules [24]. Several studies are in line with the fact that Resv, due to its hydrophobic stilbene backbone, can penetrate the lipid bilayer and interact with the acyl chains through hydrophobic forces. At low doses, Resv interacts with the polar groups, and at higher doses, Resv intercalates between the hydrophobic fatty acid chains with a broad range of angular distribution [44]. Quantum mechanical calculation has shown that the primary force driving the interaction between PC and Resv is the intermolecular hydrogen bonds between the phosphate and glycerol moieties of PC and the polyphenol’s hydroxyl groups. Additionally, Van der Waals interactions between the choline moiety and Resv also contribute to its stabilization within the membrane [45].

### 5.1. Pure PC LUVs 

The effect of a given compound on membrane structure and organization is determined by the way it interferes with the physicochemical properties of the surrounding lipids, such as their phase state. As the POPC and PDPC L_o_/L_d_ phase transition temperatures are −2.5 and −27 °C, respectively, the investigated PC LUVs exhibit a liquid-disordered (L_d_) phase state at 37 °C. The approximate location of Resv in biomembranes can be experimentally determined according to the fluorescent probe behavior response. The membrane-bound fluorescent probe Laurdan localized around the level of the glycerol backbone provides information about the degree of hydration of lipids in the bilayer at this position. In the L_d_ phase state, the membrane lipids are more loosely packed, allowing more water molecules to penetrate into the lipid bilayer. Upon Resv addition, both PC mixtures showed a decrease in membrane lipid order (fluidization) at low Resv concentrations, followed by its increase (rigidification) at all temperatures studied. The point at which the effect shifts towards rigidification for POPC membranes is 75 µM Resv (13:1 Lipid/Resv ratio), whereas for PDPC, it is about four times higher (350 µM, 3:1 Lipid/Resv ratio) at 37 °C.

Several explanations could be proposed for the Resv-induced fluidization/rigidification behavior observed in both PC mixtures. Selvaraj et al. [44] state that at low doses, Resv interacts with the phospholipid polar groups, and at higher doses, it also localizes in the hydrophobic region of POPC/POPE/Chol membranes. We assume that this intriguing phenomenon for the PC/SM/Chol mixtures may be explained in a different way. We propose that Resv can behave alternatively as a “spacer” or “filler” molecule, explaining why it can reduce or increase membrane lipid order. Resv may adopt two main distinct spatial orientations within the lipid bilayer, depending on the characteristics of its lipid environment. A position perpendicular to the fatty acid chains leads to a horizontally stretched conformation, in which Resv acts as a “spacer”. Conversely, when aligned parallel to the FA chains, the molecule behaves as a “filler”. Since PDPC membranes are more disordered compared to POPC ones, they exhibit larger and more polar space between lipid molecules. This lipid packing necessitates less energy to separate the lipids, thereby enabling more Resv to enter the bulk of lipids. A larger number of horizontally stretched conformations of Resv molecules are thus able to insert deeper into lipid bilayer. At low Resv concentrations, we propose that the decrease in lipid order observed in POPC membranes is attributable to the small number of Resv molecules in the spacer conformation. At higher concentrations, Resv acts mainly as a filler and promotes a gradual increase in the lipid order of the membrane. We assume a more favorable formation of hydrogen bonds of Resv with ester groups located deeper for PDPC compared to POPC LUVs; the latter can accommodate less Resv within the lipid bulk. This can also explain why the critical Resv concentration for reversing the lipid order is higher in PDPC than in POPC membranes. Briefly, one may assume that the distribution of hydrogen bonds between the Resv ester groups and the phosphate oxygen groups is influenced by the degree of unsaturation of the fatty acids. 

### 5.2. Binary PC/Chol Mixtures 

The role of Chol in cell membranes is critical, involving modulation of the physicochemical properties either by increasing lipid order in more disordered membranes or decreasing it in more ordered ones. Chol thus acts as a modulator of the lipid membrane’s physical properties, exhibiting varying affinities for different types of lipids and forming L_o_ domains of different sizes, depending on the lipids in the surrounding matrix. Numerous studies have investigated the interaction between Chol and PC molecules and have demonstrated a decreased affinity of Chol for PUFA-enriched PCs. In the present study, the lower Chol content in the 75/25 PC/Chol mixtures resulted in increased interaction between Resv and membranes. Conversely, in the Chol-enriched binary 50/50 mixture, Resv–membrane interaction decreased, particularly in POPC, compared to binary PDPC mixtures. It appears that high Chol levels interfere with Resv’s interaction, with glycerophospholipid membranes affecting its ordering effect on the membranes. The present results agree with those of Neves et al. [25], who show a lower partition coefficient of Resv for eggPC/Chol mixtures compared to the control (eggPC). Furthermore, Jasmin Ceja-Vega et al. [46] have found that when Chol is added to DOPC vesicles (DOPC/Chol 4:1 and 2:1 molar ratio), no significant effect of Resv on water permeability is detected. We demonstrated here that both Chol and Resv increase the membrane lipid order at the glycerol level. In low-Chol membranes, the lipid packing is less tight, allowing more Resv to be accommodated in the membrane, whereas in high-Chol membranes, the membrane is very tightly packed, with no free space between lipids for Resv insertion. This is consistent with our assumption of a greater spacer effect of Resv at low cholesterol concentrations in PDPC/Chol mixtures, whereas this spacer effect is less pronounced in mixtures containing higher Chol concentrations. At physiological temperature, SM and SM/Chol (50/50) exhibit L_β_ and L_d_ phase states and a L_o_ phase state, respectively. This spacer effect of Resv can also occur in SM-containing mixtures because Resv addition led to a slight decrease in membrane lipid order with a Resv concentration increase. 

The fact that Resv can hydrate SM/Chol (50/50) and dehydrate POPC/Chol (50/50) membranes can help in further understanding the effect of Chol in our studied mixtures. Moreover, this fact clearly demonstrates specific interactions between polyphenols and both lipid classes, glycerophospholipids and sphingolipids. The Resv dehydration effect in equimolar POPC/Chol membranes is up to four times higher compared to hydration observed in SM/Chol one. Chol makes the membrane more hydrophobic, which probably decreases the affinity of OH groups of Resv for deeper bilayer localization. Moreover, the presence of high Chol concentrations might decrease the probability of the formation of hydrogen bonds between Resv and lipids. Contrary to the generally described ordering effect of Resv described by Neves et al. [26], the addition of Resv to the equimolar SM/Chol mixture resulted in a slight dose-dependent fluidization of the L_o_ phase. A neutron reflectivity study of a mixture of PC and Chol suggests that the presence of both molecules in combination with Resv dampens the ordering effect of Chol, but still maintains the localization of Resv within the headgroups, leading to thickening of the headgroup region. Hypothetically, the formation of a nano-raft composed of a unit of PC-Resv-Chol is proposed [40].

### 5.3. Ternary PC/SM/Chol Mixtures 

PC/SM/Chol mixtures are commonly used to model plasma membranes comprising L_o_ arrangement. They mimic membrane rafts within lipids in L_d_ phase. In these mixtures, the Chol increase led to an increase in lipid order and L_o_ phase fraction. It is noteworthy that the lipid order of ternary PDPC mixtures was higher than that of POPC ones, whereas the opposite was found for pure PDPC and POPC membranes. This is not surprising since DHA has lower solubility when mixed with Chol, thus promoting the segregation of SM and Chol, leading to the formation of a larger fraction of L_o_ domains in the lipid mixtures. In POPC/SM/Chol mixtures upon Resv addition, the percentage change in lipid order increased up to 10-fold when compared to POPC alone. The monomodal ordering effect of Resv in POPC and POPC/Chol was also found to be identical for the POPC/SM/Chol mixtures. Similarly, the bimodal response of fluidizing followed by an ordering effect upon Resv addition was found for all PDPC-containing mixtures. In the ternary PDPC/SM/Chol mixture, the critical Resv concentration at which the fluidizing effect switches to more ordering is only 150 µM Resv, which is about two times lower than for binary PDPC/Chol mixtures (350 µM). The increase in Chol concentration impeded the ordering effect of Resv in ternary PDPC mixtures. 

In a previous study [33] using fluorescence microscopy and electron paramagnetic resonance (EPR), we found that ternary POPC/SM/Chol (40/40/20 and 33/33/34) mixtures have a slightly higher L_o_ fraction at 37 °C than PDPC/SM/Chol, and that this fraction increases with increasing Chol. The identification of L_o_/L_d_ coexistence depends on the detection technique used. For example, fluorescence microscopy was unable to identify micron-scale L_o_ domains in POPC/SM/Chol at physiological temperature, whereas EPR, with its nanometer-scale sensitivity, was able to detect L_o_/L_d_ phase separation in the same mixture, with L_o_ occupying up to 60% of the vesicle surface. Fluorescence microscopy was used to visualize L_o_/L_d_ phase separation in PDPC/SM/Chol mixtures. Large L_o_ micrometer domains were observed, with a percentage range of the L_o_ fraction similar to that of ternary POPC mixtures.In this study, by using DPH-TEMPO quenching, in accordance with our previous study, PDPC/SM/Chol mixtures exhibited L_o_ domains with larger sizes compared to POPC/SM/Chol ones. Furthermore, this method enabled us to determine whether Resv favors or reduces the formation of L_o_ domains. Of interest, the fluorescence probe DPH is located at the level of hydrocarbon tails of lipids, providing insight into the packing mode upon Resv addition. The addition of Resv to POPC/SM/Chol mixtures promotes the formation of L_o_ domains, the size of which gradually increases with increasing amounts of Resv. At a Chol concentration of 20 mol%, the addition of Resv resulted in a two times greater increase in L_o_ domain formation than at the higher Chol concentration of 34 mol%. This observation is consistent with a greater increase in lipid ordering by Resv at a 20 mol% Chol compared to 34 mol%. In PDPC/SM/Chol mixtures, Resv again induced a bimodal effect. The addition of Resv can either facilitate or reduce the formation of L_o_ domains, depending on the added amount. At lower concentrations of Resv, the disordering effect induced larger L_o_ domain sizes. Since Chol have a low affinity for polyunsaturated membranes, this additional decrease in membrane lipid order under Resv treatment may displace Chol from its PDPC-enriched regions, making it more available for segregation with SM, resulting in the formation of a larger L_o_ domains. 

The effect of Resv addition on the lateral L_o_/L_d_ membrane heterogeneity of the mixtures studied is consistent with Neves et al. [26]’s research on the influence of Resv on the lipid packing of liposomes composed of eggPC, Chol and SM. They showed that Resv (up to 80 µM) was able to induce phase separation, and stabilize and promote the formation of L_o_ domains in ternary mixtures. In the present study, we further refined the examination of the effect of Resv on lipid organization by using lipids with different FA chain lengths and degrees of unsaturation at the *sn-2* position, as well as by using increasing Chol concentrations. 

Upon using DLS, we established no direct correlation between nanometer LUV size and L_o_ domain size when comparing POPC and PDPC ternary mixtures, as well as be-tween the control and Resv-treated vesicles. These results are plausible considering that the DPH-TEMPO pair shows interactions in the angstrom range (6-48 Å), which is 400 to 40 times less than the size of the LUVs (150-200 nm). Thus, the effect of Resv on nanometer L_o_ domain formation is governed by changes in the physicochemical parameters of the coexisting L_o_/L_d_ phases, but not by the membrane curvature. This is in line with the conclusions made by Usery et al. [47] that the nanometer domains are characterised by complex shapes and are not considerably influenced by vesicle size.

A hydrophobic mismatch and/or a difference in the molecular order parameters of the coexisting phases are assumed to exist and explain the L_o_ and L_d_ phase segregation, with either a L_o_ thickness greater than L_d_ or a L_o_ lipid order greater than L_d_ [33,47,48]. The induced changes in the L_o_/L_d_ ratio upon Resv addition are probably attributed to its lineactant properties, which changing the hydrophobic mismatch and/or the difference in the molecular order of the coexisting phases. Regarding the bimodal behavior of the PDPC mixtures upon Resv addition, it is noteworthy that Resv can fluidize the L_d_ phase to a greater extent than the L_o_ phase in ternary PDPC mixtures. Thus, at the lower Resv dosages, the L_o_/L_d_ molecular order difference is increased, leading to an increase in line tension between the two phases. Higher Resv concentrations switch from a “spacer” mode of interaction with lipids to a “filler” one, detected by a progressive increase in the L_d_ membrane lipid order. As Resv was only able to decrease the lipid order of L_o_ in SM/Chol mixtures, it could be inferred that Resv reduces the hydrophobic mismatch between L_o_ and L_d_, thereby decreasing the driving force for L_o_ domain formation..

Considering that nanoscale phase separation is characterized by very low line tension, Usery et al. [47] suggest that in L_o_/L_d_ lipid membranes, the repulsions between dipoles dominate over the line tension, which favors the formation of numerous small domains. This nano-scale system has a considerably longer average boundary than the micron-scale system, where only one or two large domains are formed. It is possible that Resv partition in POPC/SM/Chol is more favorable at the L_o_/L_d_ interface, where the lipid packing is perturbed. Kopec et al. [49] have proposed that stilbenoids such as Resv can localize parallel to lipid tails, forming bundles that can span the entire membrane. When Resv behaves as a filler molecule, the formation of such bundles can regulate the balance between line tension and dipole repulsion, further stabilizing L_o_ domain formation in POPC/SM/Chol mixtures.

## 6. Conclusions

The present results emphasize a mode of action of biologically active molecules, such as Resv, alone and combined with omega-3-containing glycerophospholipids, on lipid membrane organization and functioning. Resv exhibits several mechanisms of action that depend on the lipid environment, including Chol, SM and FA length and unsaturation. It exerts its protective properties in a dose-dependent manner by competing with Chol or acting as a lineactant molecule. Additionally, it may adopt a filler or spacer type of intercalation in the membrane lipid bulk. The varied action of Resv on numerous biological pathways may partly be due to its impact on membrane lipid order and organization, viewed from a biophysical perspective. Resv contributes to maintaining beneficial cell functioning and defending against cardiovascular, inflammatory and cancer diseases by modulating dynamic lateral domain reorganization, membrane permeabilization and redox processes. Furthermore, by remodeling the membrane, Resv could alter numerous plasma membrane-related processes such as protein binding, sorting and signaling. It is worth noting that Resv induces endocytosis and activates down-stream pathways leading to cell death through its involvement in lipid rafts, which is consistent with its protective properties [50,51,52,53,54].

## Figures and Tables

**Table 1:** Phase transition temperature and peak width in SM control and after Resv addition.

Sample Resv (µM)	Tm (Sigmoidal Fit)	Tm (First Derivative of Sigmoid, Lorentzian Fit)	Width of Lorentzian	R^2^
0	38.5 ± 0.3	38.5	11.4	0.998
50	37.9 ± 0.4	37.9	13.8	0.998
100	37.7 ± 0.4	37.7	13.1	0.998
200	37.6 ± 0.4	37.6	14.4	0.999
500	37.4 ± 0.5	37.4	17.4	0.999

**Table 2:** Calculated domain radii (Å) in control ternary PC/SM/Chol and Resv-containing mixtures at 37 °C based on DPH-TEMPO quenching method. Values are presented as means ± SD of four independent measurements (n = 4).

Scheme 40	POPC/SM/Chol (40/40/20)	PDPC/SM/Chol (40/40/20)	POPC/SM/Chol (33/33/34)	PDPC/SM/Chol (33/33/34)
0	24.3 ± 2.2	42.9 ± 0.5	30.9 ± 0.7	43.1 ± 1.9
50	25.2 ± 1.3	43.2 ± 0.9	32.6 ± 0.9	45.2 ± 2.3
100	26.7 ± 1.2	43.3 ± 0.4	33.9 ± 1.0	45.7 ± 2.1
200	30.2 ± 1.3	42.5 ± 0.4	35.7 ± 0.8	45.0 ± 1.9
500	34.4 ± 1.1	40.7 ± 2.1	36.1 ± 1.1	44.4 ± 2.2

**Table 3:** Hydrodynamic diameter (D_h_, nm) of control PC/SM/Chol and Resv-containing LUVs at 37 °C. Mean ± SD (n = 6).

Sample Resv (µM)	POPC/SM/Chol (40/40/20)	PDPC/SM/Chol (40/40/20)	POPC/SM/Chol (33/33/34)	PDPC/SM/Chol (33/33/34)
0	173 ± 1	177 ± 4	188 ± 5	169 ± 3
100	167 ± 2	180 ± 4	169 ± 2	176 ± 3
500	165 ± 1	176 ± 1	177 ± 3	199 ± 4

**Table 4:** Polydispersity index (PDI) of control PC/SM/Chol and Resv-containing LUVs at 37 °C. Mean ± SD (n = 6).

Sample Resv (µM)	POPC/SM/Chol (40/40/20	PDPC/SM/Chol (40/40/20)	POPC/SM/Chol (33/33/34)	PDPC/SM/Chol (33/33/34)
0	0.11 ± 0.03	0.19 ± 0.03	0.19 ± 0.01	0.20 ± 0.02
100	0.16 ± 0.01	0.22 ± 0.04	0.20 ± 0.02	0.18 ± 0.04
500	0.20 ± 0.02	0.26 ± 0.03	0.25 ± 0.01	0.34 ± 0.02

## Data Availability

Data are contained within the article.
